# Factors that predict fertility desires for people living with HIV infection at a support and treatment centre in Kabale, Uganda

**DOI:** 10.1186/1742-4755-7-27

**Published:** 2010-10-11

**Authors:** Othman Kakaire, Michael O Osinde, Dan K Kaye

**Affiliations:** 1 Department of Obstetrics and Gynecology, Makerere University College of Health Sciences, P.O. Box 7072, Kampala, Uganda; 2 Kabale Regional Hospital, Department of Obstetrics and Gynecology, P.O.Box 7 Kabale, Uganda; 3 Department of Obstetrics and Gynecology, Makerere University Medical School, P.O. Box 7072, Kampala, Uganda

## Abstract

**Background:**

Studies from different contexts worldwide indicate that HIV positive patients manifest high-risk sexual behavior characterized by fertility intentions, multiple sexual partners, non-use of contraceptives and non-disclosure of HIV status to their sex partners. The objective was to analyze fertility desires among persons living with HIV at a treatment centre in Kabale Hospital, Southwestern Uganda.

**Methods:**

From January to August 2009, we interviewed 400 HIV positive patients seeking care using an interviewer-administered questionnaire. We assessed socio-demographic variables, reproductive history, sexuality and fertility desires. At bivariate and multivariate analysis, characteristics of participants who reported or did not report desire to have a child in the near future were compared.

**Results:**

Of the 400 respondents, (25.3%) were male, 47.3% were aged 25-34 years, over 85% were currently married or had ever been married, and the 62% had primary level of education or less. Over 17% had produced a child since the HIV diagnosis was made, and 28.6% reported that they would like to have a child in the near future. Age of the respondent, being single (versus being ever-married) and whether any of the respondents' children had died were inversely associated with fertility intentions.

**Conclusion:**

Factors inversely associated with fertility intentions were age of the respondent, marital status and whether any of the respondents' children had died. Use of antiretroviral therapy was not associated with fertility intentions.

## Introduction

The majority of new human immunodeficiency virus (HIV) infections that occur in children worldwide occur among children born to HIV positive mothers, who acquire the HIV infection from their mothers [1]. Through HIV treatment and support centres, HIV-infected persons and their partners are provided with the required information about the HIV prevention and treatment strategies available [2]. Despite counseling, studies from different contexts worldwide in both developed countries [3-7]and developing countries [8,9]in the era of wide access to antiretroviral drugs [10-13]indicate that many HIV positive individuals continue to exhibit high risk sexual behavior characterized by fertility intentions. Studies from Uganda indicate that 3-20% of married or cohabitating couples are HIV sero-discordant [14-16]. Within a given socio-cultural context, measuring fertility intentions among HIV-positive individuals (who were advised against having children) is a good indicator of the reproductive choices made by HIV positive individuals. The objective was to analyze fertility intentions among persons living with HIV/AIDS at a treatment and counseling centre in Kabale Hospital, Southwestern Uganda, and specifically, analyze whether being on antiretroviral therapy was associated with fertility intentions.

## Methods

### Study setting

Kabale District is a rural highland district in southwestern Uganda, about 560 km from the capital city, Kampala. The 2002 national census estimated the population of Kabale District at about 471,800, with an annual population growth rate of 3%. This district is densely populated- with an area of 1,827 square kilometers, the population density in the district in 2009 is estimated at 318 people per km². Kabale Hospital is a public regional referral hospital funded by the Uganda Ministry of Health and general care in the hospital is free.

### Study design

The study design was a cross-sectional study conducted at Kabale hospital HIV treatment and counseling centre, from January to August 2009. The study inclusion criteria were having attended for at least three months, availability of HIV sero-status results, HIV positive diagnosis and willingness to consent for participation in the study. During the eight-month study period, 400 HIV positive individuals were recruited into the study

### Data collection procedure

Participants were screened and recruited by research assistants who were health workers attached to the HIV treatment, care and support centre. Using an interviewer-administered questionnaire and through records review of information in the client database, data was collected on socio-demographic variables such as age, education level, marital status, number of years in marriage, employment status, and social habits (drinking alcohol or smoking). Reproductive history: parity and number of living children(number of biological children of the respondent who were alive), sexuality (number of sexual partners, change of sexual partners, frequency of sexual intercourse and condom use) were also assessed. For fertility intention, individuals' subjective feelings regarding future conception (whether the respondent wanted to have a child in the next two years) was assessed.

### Data analysis

Data was analyzed using the STATA software (Release 9) to provide frequencies and percentages for categorical variables and means and standard deviations for numerical variables. At bivariate analysis, characteristics of the participants who reported fertility intentions were compared with those who did not, using *Pearson's *chi-square test for categorical data and Student *t*-test for numerical data. To adjust for confounding, collinearity and interaction and thereby analyze factors that were independently associated with fertility intention, multivariate logistic-regression analysis was conducted. During the stepwise modeling for regression analyses, all variables of clinical importance or with p-value 0.2 and less on bivariate analysis were considered for inclusion. Intention to have a child in the near future (fertility intention) was entered as intends* = 1, does not intend = 0*. Ever treatment for sexually transmitted infection since the HIV diagnosis was entered as Ever treated * = 1, Never treated = 0*. Consistent contraceptive use was entered as *present = 1, absent = 0*. Parity, number of living children and age of spouse, age of sexual debut and age at marriage were entered as numerical variables. Other categorical variables were entered as *present or yes = 1*, and *absent or No *= 0. Participant age was evaluated as a numerical variable and as 5-year age categories. The model goodness-of-fit of the final logistic regression models was assessed by Pearson's chi-square test.

### Ethical considerations

Ethical approval was obtained from Kabale Regional Hospital. Counseling about sexuality, condom use, dual protection and fertility was provided to all the participants, and all eligible participants were provided with antiretroviral therapy.

## Results

Of the 400 respondents (Table 1), 101 (25.3%) were male, 47.3% were in the age category 25-34 years, over 85% were currently married or had ever been married, and 62% had primary level of education or less. The mean age of sexual debut, age at marriage and age when participants had their first child was 18.3 years, 20.5 years and 21.8 years respectively. Only 191 (53.1%) described their intimate relationships as stable.

**Table 1:** Socio-demographic characteristics of the study participants

Characteristic	Number (Percentage) or Mean (± Standard deviation)
*Sex*	
Males	101 (25.3)
Females	299 (74.7)

*Age category*	
Less than 24 years	55 (13.8)
25-29 years	106 (26.5)
30-34 years	79 (19.8)
35-39 years	53 (13.3)
40-44 years	37 (9.3)
45-49 years	51 (12.8)
50 years or more	20 (5.1)

*Religion*	
Catholic	140 (35.3)
Protestant	205 (51.2)
Moslem	32 (8.0)
Others	23 (5.0)

*Do you have stable sexual relationship?*	
Yes	191 (53.1)
No	159 (46.9)

*Marital status*	
Single	59 (14.8)
Married	196(49.0)
Widow/widowed	111(27.8)
Divorced or separated	34 (8.4)

*Level of education*	
No formal education	58 (14.8)
Primary	188 (47.1)
Secondary	124 (31.1)
Tertiary or university	30 (7.9)

*Drink alcohol*	
Yes	93(23.4)
No	307 (76.5)

*Have any of your children died?*	
Yes	158(39.5)
No	242 (60.5)

Number of living children	3.0 ± 1.4

Mean age of sexual debut	18.3 ± 2.9

Mean age at first marriage if married	20.5 ± 3.8

Mean age when participants had first child	21.8 ± 3.9

Table 2 shows the reproductive history of the respondents stratified by gender. Of the 400 respondents, 63 (17.6%) had produced a child since the HIV diagnosis was made, and 108 (28.6%) reported that they intended to have a child in the near future (within next two years). Fifty four respondents (13.4%) were unaware that if they were HIV positive, they could pass on the virus to the unborn baby. Only 214 (53.1%) reported that they were aware that their regular sexual partner/partners knew their HIV sero-status, despite 330 respondents (82.5%) already taking antiretroviral drugs. Of the 197 (49.3%) who were aware of the partners' HIV sero-status, 170 (86.7%) knew the partners were HIV positive. More than 10% had had sexual intercourse with more than one sexual partner in the previous 6 months.

**Table 2:** Reproductive and sexual history of the study participants stratified by gender

Characteristic	Males; Number (Percentage)	Females; Number (Percentage)
*Are you currently using contraceptives		
Yes	61 (60.4)	149 (40.5)
No	29 (39.6)	141 (49.8)

*Do you have stable sexual relationship?		
Yes	62 (31.7)	129 (43.1)
No	35 (69.3)	133 (56.9)

*Have you had sex in the last 3 months?		
Yes	74 (38.2)	163 (54.5)
No	26 (41.8)	133 (45.5)

*On average, how often did you have sex in the last 3 months?		
At least 3 times per week	15 (14.9)	32 (10.7)
Around once a week	38 (37.6)	82 (27.4)
About once a month	22 (21.8)	47 (15.7)
Less frequently than once a month	16 (15.8)	115 (38.5)

*How many sexual partners have you had in the last 6 months?		
None	22 (21.8)	113 (37.8)
One	59 (58.4)	158 (52.8)
Two	10 (10.5)	10 (3.3)
Three	5 (5.0)	4 (1.3)
More than 3	2 (2.0)	7 (2.3)

*Does your regular partner know your HIV status?		
Yes	68 (67.3)	146 (48.8)
No	19 (32.7)	57 (51.2)

*Are you currently is on ARVs?*		
Yes	93 (92.1)	235 (78.6)
No	8 (7.9)	64 (21.4)

*Is your partner HIV positive?		
Yes	57 (90.5)	112 (84.2)
No	6 (9.5)	21 (15.8)

*Have you changed regular sexual partners since HIV diagnosis?		
Yes	39 (38.6)	105 (35.1)
No	62 (61.4)	210 (64.9)

Key- * Non response was partly due to some respondents were not sexually active or did not have sexual partners

In table 3, having produced a child since the HIV diagnosis, being single (versus being married, separated or divorced), the number of living children, whether the respondent perceived the relationship as stable and having had sex in the previous six months were significantly associated with fertility intention (though respondents reported less likelihood of desiring to have children in the next two years) (p-value less than 0.05). On the other hand, the number of sexual partners in the previous six moths was significantly associated with fertility intention (those with more sexual partners were more likely to have fertility intention (p-value less than 0.05)). Being on ARVs was not significantly associated with fertility intentions. In table 4, being on antiretroviral therapy was not significantly associated with fertility intentions. The factors independently associated with desiring a child in the near future among HIV positive persons were age of the respondent, marital status and when any of the respondents' children had died. Young age, those who were single (as compared to the ever-married) and respondents whose children had died were significantly less likely to have fertility intentions.

**Table 3:** Bivariate analysis of factors associated with fertility intentions among HIV positive individuals attending the HIV care and support centre.

Characteristic	Odds ratio and confidence limits	p-value
*Sex*		
Male versus Female (reference group)	1.80 (1.00, 3.24)	0.050

Age of respondent	0.56 (0.46, 0.67)	< 0.001

Age at sexual debut	0.98 (0.90, 1.06)	0.558

Religion	0.92 (0.70, 1.22)	0.574

*Marital status*		
Single versus married, divorced or separated	0.54 (0.41, 0.72)	< 0.001

Occupation	0.92 (0.79, 1.07)	0.292

Drinking habits Drinks versus does not drink	0.65 (0.39,1.09)	0.107

Number of living children	0.83 (0.70, 0.97)	0.027

Whether any of the respondents' children died Yes versus No	0.97 (0.61, 1.54)	0.891

Whether respondent is on ARVs Yes versus No	1.52 (0.87, 2.66)	0.142

Whether respondent had sex in previous 6 months Yes versus No	0.38 (0.62, 0.92)	< 0.001

Number of sexual partners in the previous 6 months	1.31 (1.01, 1.71)	0.041

Has the respondent disclosed HIV status to sexual partner/partners? Yes versus No	0.81 (0.45, 1.4)	0.478

Are any of respondent's sexual partners HIV positive? Yes versus No	1.00 (0.76, 1.34)	0.953

Has participant changed sexual partners in previous 3 months? Yes versus No	0.69 (0.42, 1.12)	0.146

Is the respondent currently using contraceptives? Yes versus No	0.75 (0.48, 1.20)	0.226

Is the respondent using dual protection? Yes versus No	0.96 (0.75, 1.20)	0.753

Since the HIV diagnosis, has the respondent produced a child? Yes versus No	0.40 (0.23, 0.69)	0.001

When does the respondent you use condoms? With regular partners versus with casual partners	0.89 (0.52, 1.53)	0.673

**Table 4:** Factors among the HIV positive participants that independently predict fertility intentions

Covariate	Odds ratio and confidence limits	p-value
Age of respondent	0.55 (0.43, 0.71)	< 0.001

Sex of respondent	1.10 (0.51, 2.25)	0.861

Marital status Single versus ever married (married, separated or widowed)	0.61 (0.41, 0.90)	0.012

Number of living children	0.99 (0.79, 1.24)	0.946

Are any of the respondents' children dead? Yes versus No	0.48 (0.26, 0.88)	0.018

Is respondent is currently on ARVs? Yes versus No	1.13 (0.57, 2.27)	0.724

Has the respondent had sex in previous 6 months? Yes versus No	0.48 (0.21, 1.07)	0.075

Average number of sexual partners Any number versus None	1.07 (0.80, 1.44)	0.635

Has respondent changed partners since HIV diagnosis? Yes versus No	0.84 (0.45, 1.54)	0.571

## Discussion

Since access to antiretroviral therapy has improved quality of life and survival for HIV infected people, many will contemplate child bearing. Identification of contextual determinants of decision to have children among HIV positive couples is useful for designing of policies and establishing intervention priorities in reproductive health for this population. Being on antiretroviral therapy was not associated with fertility intention in this population. Respondents who were of young age, were single or had lost a child were unlikely to have fertility intentions.

Several studies that have described pregnancy intention rates in different contexts of people living with HIV have reported rates that range from 17% in Uganda [10] to 63% in Nigeria [17]. Studies from Zambia [18] and Zimbabwe [19] found no effect of HIV diagnosis on subsequent childbearing, implying that pregnancy intentions were unaffected by HIV diagnosis. Fertility intentions among HIV positive persons are more common in developing countries (where the overall fertility in the population is still high, contraceptive use is low, and unmet need for contraception is high). They have also been noted in more developed countries such as Brazil, United States of America, France and Italy [20-26].

The finding of lack of association of fertility intention and antiretroviral therapy contrasts that of Maier et al [13] in the same socio-cultural context who had found that antiretroviral therapy had increased fertility desires (but had little influence on pregnancy rates and live birth rates of HIV positive individuals). At the time when the HIV pandemic in the developing countries matures, the majority of affected individuals are in the reproductive age, where they may desire to have children in spite of their HIV status [10,15,27]. Factors influencing HIV positive people's pregnancy decisions, especially partner's desire to have a child and young age do not differ from those of influencing HIV non-infected women [28].

The study findings are consistent with previous research which showed that many cohabiting couples do not mutually know their HIV status [10,13,29]. HIV prevention programs to protect the negative partners in discordant couples in Uganda face the dilemma of non-disclosure of HIV status for HIV positive individuals [14,16], many of whom are already taking antiretroviral drugs. Some of the contextual factors that influence sexuality, fertility intentions, reproductive decision making and subsequent sexual behavior of HIV positive individuals are personal and partners' desire to have children, societal reproductive expectations and non-disclosure of HIV sero-status in sexual relationships [14,16,29]. These contextual factors, which seem not to have changed for HIV positive individuals in the era of access to antiretroviral therapy, might be partly responsible for the high unmet need for contraception [10,30,31].

## Conclusion

Factors that influence fertility intention among HIV positive persons were age of the respondent, marital status and whether any of the respondents' children had died. Respondents who were of young age, were single or had lost some children were unlikely to desire a child in the near future. Being n antiretroviral therapy was not associated with desire to have a child in the near future. The finding that counseling and support given from an HIV care and treatment center did not alter HIV positive individuals' perceptions and subsequent sexual behavior has implication for counseling these clients.

## Competing interests

The authors declare that they have no competing interests.

## Authors' contributions

MOO and OK conceptualized the study. DKK, OK and MO designed the study instrument. MOO collected the data. DKK and OK conducted the data analysis. DKK wrote the first draft of the manuscript. All co-authors contributed to revision of the subsequent draft manuscripts and approved the final and this version of the manuscript.
